# The relationship between daytime sleepiness and working memory performance in Russian adolescents: evidence from the Backward Digit Span task

**DOI:** 10.3389/fpsyg.2026.1748332

**Published:** 2026-05-20

**Authors:** Tian Xinxin, Valeriia Demareva, Artem Malykh, Wang Xinghua, Victoria Ismatullina, Timofey Adamovich, Sergey Malykh

**Affiliations:** 1 Faculty of Russian Language, Harbin University of Science and Technology, Harbin, China; 2 Cyberpsychology Laboratory, Faculty of Social Sciences, Lobachevsky State University of Nizhny Novgorod, Nizhny Novgorod, Russia; 3 Center of Population Research, Ural Institute of Humanities, Ural Federal University, Ekaterinburg, Russia; 4 Oriental Language Institute, Mudanjiang Normal University, Mudanjiang, China; 5 Developmental Behavioral Genetics Laboratory, Psychological Institute of Russian Academy of Education, Moscow, Russia

**Keywords:** adolescence, Backward Digit Span, cognitive performance, daytime sleepiness, executive function, PDSS, verbal cognition, working memory

## Abstract

Daytime sleepiness is a common experience during adolescence and has been linked to decrements in cognitive performance. However, evidence regarding its association with verbal working memory remains limited. The present study examined whether higher daytime sleepiness, as measured by the Pediatric Daytime Sleepiness Scale (PDSS), is related to lower working memory performance in adolescents. A total of 880 adolescents (436 males, 444 females; *M*_age_ = 14.5, *SD* = 1.4) completed the PDSS and a computerized Backward Digit Span (BDS) task assessing verbal working memory. The main outcome measures were the longest correctly recalled sequence and the number of correct responses. Linear regression models were used to test whether PDSS predicted BDS performance while controlling for age and sex. Additional analyses compared participants with Low (PDSS < 16) and High (PDSS ≥ 16) levels of daytime sleepiness. Higher PDSS scores were associated with shorter Backward Digit Span sequences (*β* = −0.043, *p* = 0.004) and fewer correct responses (*β* = −0.035, *p* = 0.052). Adolescents with high PDSS scores performed significantly worse than their low-PDSS peers (*β* = −0.43, *p* = 0.027). Age positively predicted BDS outcomes, and females outperformed males. Daytime sleepiness showed a small but consistent negative relationship with verbal working memory capacity in adolescence. These findings suggest that even moderate increases in daytime sleepiness may reduce the efficiency of cognitive control processes during this developmental period.

## 1 Introduction

Adolescence is a developmental period marked by dramatic changes in sleep–wake regulation, neural maturation, and cognitive performance. During this stage, many adolescents experience insufficient or irregular sleep, often resulting in increased daytime sleepiness (e.g., Zucco et al., 2025). Daytime sleepiness—defined as the propensity to fall asleep or feel drowsy during waking hours—has been linked to memory (Ding et al., 2023; Malykh and Demareva, 2025) and attention deficits (Loram et al., 2021), impairments in complex cognitive functions (Macchitella et al., 2020), and poorer academic outcomes (Fredrick et al., 2023), a relationship partly mediated by alterations in brain morphology (Guo et al., 2025). Several mechanisms have been proposed to explain these associations. Daytime sleepiness has been linked to reduced sustained attention, diminished cognitive control, and lower efficiency of prefrontal executive networks, which play a key role in working memory manipulation processes (Alsameen et al., 2021; Barel and Tzischinsky, 2022; Ding et al., 2023). Yet, the specific cognitive systems affected by elevated sleepiness remain under-researched in normative adolescent samples.

Of particular interest is working memory, the capacity to hold and manipulate information over short periods, which underpins reasoning, learning, and academic achievement. Experimental and neuroimaging studies demonstrate that sleep restriction impairs working memory, especially when manipulation (not just storage) is required (Alsameen et al., 2021). However, while these studies focus on acute sleep manipulation and small samples, there is limited evidence on how habitual daytime sleepiness relates to working memory performance in real-world adolescent populations.

The Backward Digit Span (BDS) task is a widely used measure of working memory in children (St Clair-Thompson, 2010; Hilbert et al., 2015) that places demands on both maintenance and manipulation of information. Because BDS involves reversing presented sequences, it may be sensitive to reduced alertness or fatigue states, which impair executive control. Empirical evidence from adolescent populations suggests that poorer sleep quality and shorter sleep duration are associated with lower BDS scores (Wang et al., 2022), yet the role of self-reported daytime sleepiness remains under-examined.

Subjective daytime sleepiness is typically measured using scales such as the Pediatric Daytime Sleepiness Scale (PDSS). Recent population-based research shows that higher PDSS scores are associated with poorer alertness and cognition in adolescents (Li et al., 2025). Given that adolescence is characterised by both increasing daytime sleepiness and evolving cognitive capacity (Barel and Tzischinsky, 2022), it is important to understand how these two trajectories might intersect.

The present study aims to examine the relationship between daytime sleepiness and verbal working memory performance in adolescence, using the PDSS and a computerized BDS task. We hypothesise that higher PDSS scores will predict poorer BDS performance (shorter sequences and fewer correct answers), even after controlling for age and sex. Additionally, we anticipate that adolescents classified with elevated daytime sleepiness (PDSS ≥ 16) will perform worse on BDS than those with lower sleepiness.

## 2 Method

### 2.1 Participants

The study included 880 adolescents (436 males coded as 1, 444 females coded as 2) aged between 12 and 18 years (*M* = 14.5, *SD* = 1.4). Participants were recruited from several secondary schools as part of a larger project on daytime sleepiness and cognitive performance in adolescence.

All study procedures complied with the ethical principles outlined in the 1964 Declaration of Helsinki and its subsequent amendments. Prior to data collection, written informed consent was obtained from the legal guardians of all participants. Adolescents were informed about the aims of the study and participated on a voluntary basis. The research protocol received ethical approval from the Ethics Committee of the Psychological Institute of the Russian Academy of Education (Protocol No. 2020/4-1, approved April 2, 2020).

### 2.2 Measures

#### 2.2.1 Daytime sleepiness

Daytime sleepiness was assessed using the Pediatric Daytime Sleepiness Scale (PDSS), which has been previously validated for use in Russian adolescent samples (Zakharov et al., 2023). The instrument demonstrated satisfactory internal consistency (Cronbach’s *α* ≈ 0.78). It comprises eight items rated on a five-point Likert scale ranging from 0 (never) to 4 (always), yielding a total score between 0 and 32, where higher values reflect greater daytime sleepiness.

Based on established criteria, scores were dichotomized into normal and high sleepiness groups using a cutoff of 16, applicable to both sexes (Meyer et al., 2017; Kolomeychuk et al., 2019). Participants completed the questionnaire independently during school hours without parental verification, which is acknowledged as a methodological limitation. In the present sample, the mean PDSS score was 13.7 (*SD* = 6.5).

#### 2.2.2 Backward Digit Span (BDS)

Verbal working memory was measured using a computerized Backward Digit Span. On each trial, participants viewed random digit sequences (3–10 digits), each shown for 500 ms with a 250 ms blank interval. They were instructed to type the digits in reverse order in a response field. The sequence length increased after each correct response and decreased after an incorrect one. After three consecutive errors, the task ended automatically.

Two performance indices were computed:

1. Longest sequence length (BW_LongestSequence)—the longest correctly reproduced digit sequence,
2. Number of correct responses (BW_CorrectAnswers)—the total number of correct trials. Both indices reflect verbal working memory capacity and manipulation efficiency.

### 2.3 Procedure

Testing took place individually in quiet computer classrooms during regular school hours between 9:00 and 13:00.

Participants first completed the Backward Digit Span task, followed by the PDSS questionnaire. Each session lasted approximately 15–20 min. Instructions emphasized accuracy and sustained attention throughout the task.

### 2.4 Data analysis

All analyses were conducted in RStudio software (2024.04.2 Build 764). Cases with missing data in PDSS, BDS, age, or sex were excluded listwise, yielding the final sample (*N* = 880). Descriptive statistics and Pearson correlations were computed for PDSS, age, and BDS measures. To test whether daytime sleepiness predicted working memory performance, two sets of linear regression models were fitted:

1 Continuous PDSS models (A–B):

  PDSS total score entered as a continuous predictor with Age and Sex (m = male, f = female) as covariates. Dependent variables: *BW_LongestSequence* (Model A) and *BW_CorrectAnswers* (Model B).

2 Categorical PDSS models (D–E):

PDSS dichotomized at 16 points (Low PDSS < 16; High PDSS ≥ 16). Age and Sex were again included as covariates.

All analyses used two-tailed tests with *α* = 0.05. Regression results are reported with standardized *β* coefficients, *p*-values, and *R^2^* indices. Effect sizes for group comparisons were estimated as Cohen’s *d*.

Prior to conducting parametric analyses, the distribution of the main variables was examined using visual inspection of histograms and Shapiro–Wilk tests. The distributions did not deviate substantially from normality, supporting the use of parametric statistical methods.

## 3 Results

### 3.1 Descriptive statistics

The final sample consisted of 880 adolescents aged 12–18 years (*M* = 14.5, *SD* = 1.4).

Sex distribution was balanced (49% males, 51% females).

Daytime sleepiness (PDSS) ranged from 0 to 32 (*M* = 13.72, *SD* = 6.53).

Backward Digit Span (BDS) performance was moderate, with a mean longest sequence length of 6.57 ± 2.93 and correct answers 6.51 ± 3.54.

Across age groups, most participants were 13–15 years old (Table 1).

**Table 1:** Descriptive statistics of the sample and main variables.

Variable	M	SD	Min	Max
Age (years)	14.5	1.4	12	18
PDSS total	13.7	6.5	0	32
BW LongestSequence	6.57	2.93	1	14
BW CorrectAnswers	6.51	3.54	0	19

Using a PDSS cut-off of 16 points, 43% of adolescents were classified as High PDSS (elevated daytime sleepiness).

Exploratory analyses testing the interaction between PDSS and age did not reveal significant effects for either Backward Digit Span outcome (all *p* > 0.60), indicating that the association between daytime sleepiness and working memory performance did not differ across age.

**Figure 1:**
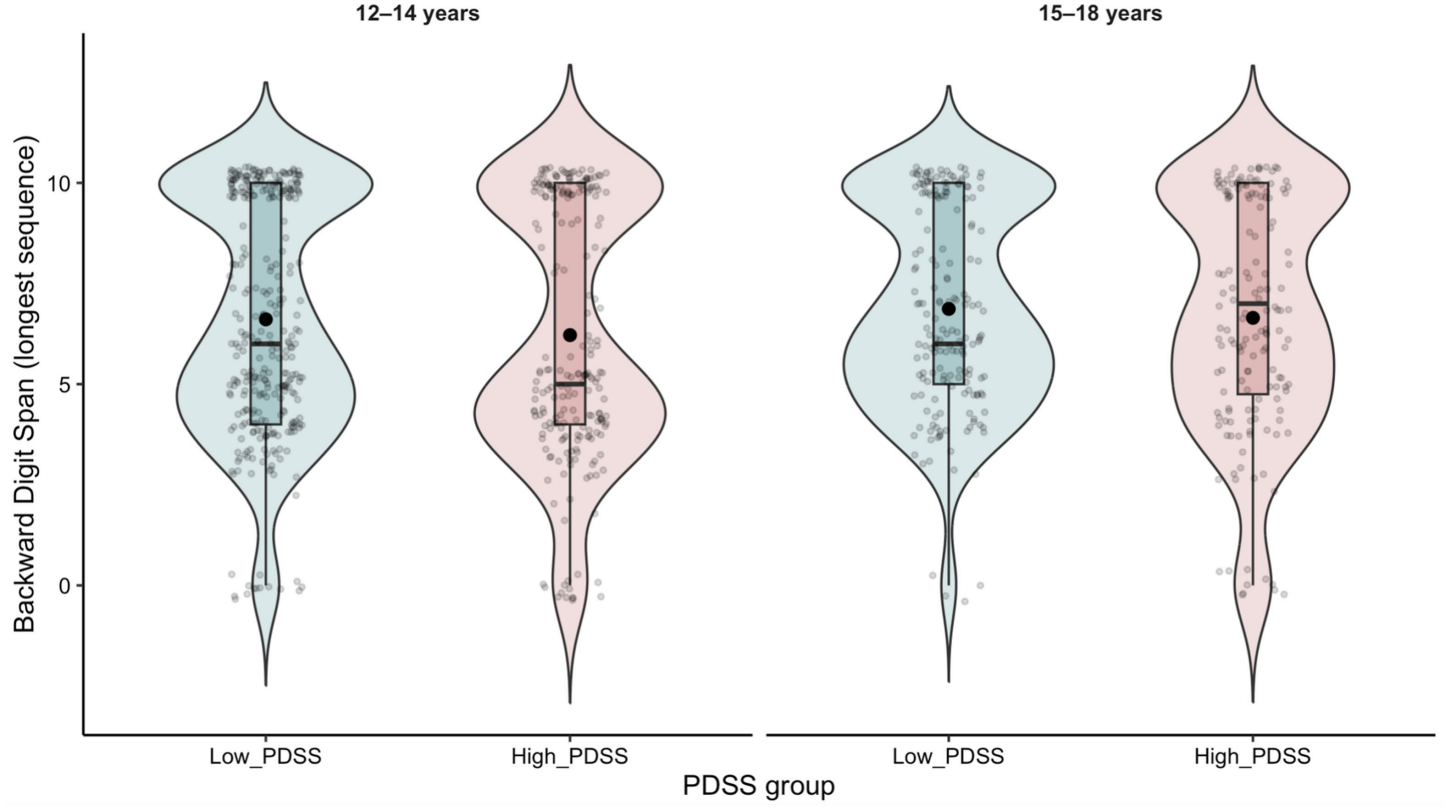
Distribution of Backward Digit Span performance across daytime sleepiness groups, shown separately for younger (12–14 years) and older (15–18 years) adolescents. Violin plots represent score distributions, boxplots indicate the median and interquartile range, and individual observations are shown as jittered points. Black dots indicate group means.

In addition, we included a distribution plot illustrating Backward Digit Span performance across PDSS groups separately for younger and older adolescents (Figure 1).

### 3.2 Correlation between PDSS, working memory performance, and age

Pearson correlations (Table 2) showed that daytime sleepiness was negatively related to working memory performance, while age was positively related to both Backward Digit Span measures.

**Table 2:** Pearson correlations among PDSS, age, and Backward Digit Span variables.

Variable	1	2	3	4
1. PDSS total	—			
2. Age	−0.04	—		
3. BW LongestSequence	−0.11**	0.21***	—	
4. BW CorrectAnswers	−0.09*	0.25***	0.86***	—

*p < 0.05; **p < 0.01; ***p < 0.001.

Higher PDSS scores were associated with shorter backward sequences (*r* = −0.11, *p* < 0.01) and fewer correct responses (*r* = −0.09, *p* < 0.05). In contrast, age correlated positively with both the longest sequence (*r* = 0.21, *p* < 0.001) and the number of correct answers (*r* = 0.25, *p* < 0.001), indicating developmental improvement in working memory across adolescence. Correlations between PDSS and age were not significant (*r* = −0.04, *p* > 0.10), suggesting that the effect of sleepiness is not simply age-dependent.

### 3.3 Regression models with PDSS score

Linear regression analyses were conducted to examine whether daytime sleepiness, measured by PDSS, predicted Backward Digit Span (BDS) performance after controlling for age and sex.

Results showed that higher PDSS scores were associated with poorer performance on both BDS indicators (Table 3).

**Table 3:** Linear-regression models predicting Backward Digit Span performance from PDSS (continuous).

Model	Dependent variable	*R* ^2^	Predictor	*β*	SE	*p*
A	BW LongestSequence	0.063	PDSS (total)	−0.043	0.015	0.004
			Age	0.207	0.069	0.003
			Sex (f vs. m)	1.22	0.20	<0.001
B	BW CorrectAnswers	0.047	PDSS (total)	−0.035	0.018	0.052
			Age	0.33	0.08	<0.001
			Sex (f vs. m)	1.03	0.24	<0.001

In the model predicting the longest backward sequence (Model A), PDSS was a significant negative predictor (*β* = −0.043, *p* = 0.004), indicating that each additional point of daytime sleepiness corresponded to a decrease of approximately 0.04 digits in maximum sequence length.

Age showed a significant positive association (*β* = 0.21, *p* = 0.003), suggesting developmental improvement in working memory capacity across adolescence. Sex differences were also observed, with females outperforming males (*β* = 1.22, *p* < 0.001). The model explained about 6% of the variance in performance (*R^2^* = 0.063).

A similar pattern emerged in the model predicting the number of correct responses (Model B).

Here, the PDSS effect approached significance (*β* = −0.035, *p* = 0.052), and both age (*β* = 0.33, *p* < 0.001) and sex (*β* = 1.03, *p* < 0.001) remained significant predictors. Although the effect of PDSS was smaller in this model, the direction of association was consistent, reinforcing the negative link between daytime sleepiness and working memory efficiency.

### 3.4 Regression models with PDSS levels

To further examine the relationship between daytime sleepiness and verbal working memory, participants were divided into two groups based on their PDSS scores: Low PDSS (<16 points) and High PDSS (≥16 points). This cut-off has been commonly used in adolescent sleep research to indicate elevated daytime sleepiness. Regression analyses were then performed using this categorical factor while controlling for age and sex (Table 4).

**Table 4:** Linear-regression models with PDSS group (cut-off = 16) predicting Backward Digit Span performance.

Model	Dependent variable	*R* ^2^	Predictor	*β*	SE	*p*
D	BW LongestSequence	0.060	PDSS group (High vs. Low)	−0.43	0.20	0.027
			Age	0.21	0.07	0.003
			Sex (f vs. m)	1.18	0.19	<0.001
E	BW CorrectAnswers	0.046	PDSS group (High vs. Low)	−0.46	0.24	0.055
			Age	0.33	0.08	<0.001
			Sex (f vs. m)	1.01	0.24	<0.001

Results revealed that adolescents with high PDSS scores performed significantly worse on the Backward Digit Span task. In the model predicting the longest backward sequence (Model D), the effect of PDSS group was statistically significant (*β* = −0.43, *p* = 0.027). This indicates that, on average, participants with elevated daytime sleepiness reproduced shorter sequences—approximately half a digit less—than their less sleepy peers, even after accounting for age and sex. Age remained a significant positive predictor (*β* = 0.21, *p* = 0.003), and females again outperformed males (*β* = 1.18, *p* < 0.001). The model explained about 6% of the variance in performance (*R^2^* = 0.060).

A similar tendency was observed for the number of correct answers (Model E).

Although the PDSS-group effect only approached significance (*β* = −0.46, *p* = 0.055) and the effect size was small (Cohen’s *d* ≈ 0.11), the pattern was consistent across both the continuous and categorical models, the direction of the relationship was consistent with the continuous models, supporting the robustness of the link between higher daytime sleepiness and poorer working memory performance.

## 4 Discussion

This brief report examined whether adolescents who report higher daytime sleepiness also show lower performance on a verbal working memory task. Our hypotheses were confirmed: daytime sleepiness negatively predicted working memory performance, both when treated as a continuous variable and when participants were grouped by PDSS cut-off scores. Across correlational and regression analyses, daytime sleepiness (PDSS) was consistently associated with poorer Backward Digit Span (BDS) performance, with small but reliable effects. The pattern held when PDSS was modeled both continuously and categorically (cut-off = 16), and after adjusting for age and sex. In line with developmental expectations, older adolescents performed better on BDS, and females outperformed males. Together, these findings indicate that even moderate increases in daytime sleepiness are linked to reduced efficiency of verbal working memory in adolescence.

The proportion of adolescents classified as having elevated daytime sleepiness (43%) is comparable with estimates reported in several adolescent sleep studies, where prevalence rates between approximately 35 and 55% have been reported (Chung and Cheung, 2008; Lund et al., 2010; Meyer et al., 2019).

Our results add to a growing literature documenting cognitive correlates of adolescent sleepiness. Although the correlations were relatively small, such effect sizes are typical in individual-differences research and large population-based samples (Gignac and Szodorai, 2016). Even modest associations may have meaningful implications at the population level. Prior work has associated insufficient or irregular sleep and subjective sleepiness with memory and attention difficulties and broader executive inefficiencies (Macchitella et al., 2020; Loram et al., 2021; Ding et al., 2023; Malykh and Demareva, 2025). Longitudinal and mechanistic accounts also connect evening preference and sleepiness with academic impairment (Fredrick et al., 2023) and, in large samples, with population-level trajectories of sleep problems (Zucco et al., 2025). Within the executive domain, experimental and neuroimaging studies show that sleep restriction disproportionately hampers tasks that require manipulation, not just storage, of information (Alsameen et al., 2021), which aligns with the backward (vs. forward) span being particularly sensitive to alertness fluctuations. Consistent with this view, studies relating sleep parameters to digit-span outcomes report lower performance with shorter sleep or poorer sleep quality (Wang et al., 2022), and adolescent work links daytime nap or rest to improvements in attention/working memory measured with backward span (Zare et al., 2023). Our findings extend these observations by showing that self-reported daytime sleepiness (PDSS) relates to BDS performance in a large, ecologically valid school sample.

Two features strengthen the interpretation. First, the convergent pattern across continuous and categorical PDSS models suggests robustness to the way sleepiness is operationalized, echoing proposals for practical cut-offs (Meyer et al., 2017) and regional data on PDSS distributions (Kolomeychuk et al., 2019). Second, we used a validated Russian PDSS adaptation with acceptable internal consistency (Randler et al., 2019), improving measurement comparability. Conceptually, the link between PDSS and BDS fits with accounts that tie sleepiness to attention control, processing efficiency, and maintenance/manipulation dynamics in working memory (St Clair-Thompson, 2010; Hilbert et al., 2015; Barel and Tzischinsky, 2022; Ding et al., 2023). Although effect sizes were small, such magnitudes are typical in population-based adolescent studies and may carry meaningful educational implications at scale (e.g., cumulative classroom impact, test performance variability).

At the same time, several limitations warrant caution. The design is cross-sectional, precluding causal inference: higher PDSS may contribute to poorer working memory, but lower cognitive control could also exacerbate sleep habits that raise daytime sleepiness. PDSS is a self-report measure; objective indices (e.g., actigraphy, polysomnography) and diary-based sleep regularity were not collected. We focused on a single task (BDS); while well-established for working memory (St Clair-Thompson, 2010; Hilbert et al., 2015), a broader battery would clarify domain specificity. Potential confounds such as chronotype, ADHD symptoms, or screen time—each associated with sleepiness and cognition—were not modeled here (Richardson et al., 2018; Loram et al., 2021; Soares et al., 2021; Fredrick et al., 2023). Finally, classroom administration lacked parental verification of responses, which we acknowledge as a methodological constraint.

These limitations suggest clear avenues for future work. Longitudinal or intervention designs (e.g., sleep hygiene, delayed school start times, or brief nap opportunities) could test whether reducing daytime sleepiness yields measurable gains in working memory. Incorporating objective sleep and chronotype assessments, as well as attention and processing-speed measures, would help disentangle whether PDSS effects on BDS reflect central executive constraints, sustained attention lapses, or more general processing-speed reductions (Alsameen et al., 2021; Barel and Tzischinsky, 2022; Ding et al., 2023). Finally, examining academic endpoints (grades, standardized tests) would bridge cognitive effects with outcomes of direct educational relevance (Fredrick et al., 2023; Guo et al., 2025; Li et al., 2025).

Future research may also examine genetic contributions to individual differences in sleep regulation and cognitive performance.

In conclusion, adolescents reporting greater daytime sleepiness show modest but consistent decrements in verbal working memory performance on the Backward Digit Span. Given the central role of working memory in learning, our findings support routine monitoring of daytime sleepiness (e.g., PDSS screening) and school-based prevention targeting sleep regularity. Even small improvements in alertness may translate into functionally meaningful benefits for cognitive efficiency and educational engagement at the population level.

## Data Availability

The datasets presented in this article are not readily available because the datasets analyzed during the current study contain confidential information and cannot be made publicly available due to institutional regulations. Access to the anonymized datasets may be granted by the corresponding author upon request. Applicants will be required to describe the intended purposes of data use. Requests will be reviewed by the institutional ethics committee, and applicants will need to justify the use of the dataset within the framework of Open Science and sign a waiver prohibiting its use for commercial purposes. Requests to access the datasets should be directed to Sergey Malykh malykhsb@mail.ru.
